# Effects of Different Air Particle Abrasion Protocols on the Biaxial Flexural Strength and Fractography of High/Ultra-Translucent Zirconia

**DOI:** 10.3390/ma15010244

**Published:** 2021-12-29

**Authors:** Reem AlMutairi, Hend AlNahedh, Ahmed Maawadh, Ahmed Elhejazi

**Affiliations:** Department of Restorative Dental Sciences, College of Dentistry, King Saud University, P.O. Box 93504, Riyadh 11683, Saudi Arabia; HAlNahedh@ksu.edu.sa (H.A.); amaawadh@ksu.edu.sa (A.M.); ahmed.hejazi@gmail.com (A.E.)

**Keywords:** airborne particle abrasion, biaxial flexural strength, fractography, high translucent zirconia

## Abstract

In this study, the biaxial flexural strength (BFS) and fractography of high/ultra-translucent monolithic zirconia ceramics subjected to different mechanical surface pretreatments were evaluated. A total of 108 disc-shaped samples (12 mm diameter, 1.2 mm thickness) of three zirconia materials (5Y-ZP KATANA Zirconia UTML (ML), 3Y-TZP DD Bio ZX2 (DB), and 5Y-ZP DD cube X2 (DC)) were used. The BFS was investigated after subjecting the samples to surface treatment using air abrasion particles of two types (aluminum oxide or glass microbeads). The data were analyzed using two-way analysis of variance, followed by Scheffe’s post hoc test for multiple comparisons. The mean ± standard deviation BFS for DB was highest after treatment with 50 µm Al_2_O_3_ (1626.05 ± 31.9 MPa), with lower values being observed following treatment with 50 µm glass microbeads (1399.53 ± 24.2 MPa) and in the control sample (1198.51 ± 21.1 MPa). The mean ± standard deviation (SD) BFSs for DC and ML were the highest in the control groups. Surface air abrasion with 50 µm Al_2_O_3_ particles and 2 bar pressure is recommended for 3Y-TZP translucent zirconia, while no abrasion of 5Y-ZP translucent zirconia ceramic.

## 1. Introduction

All-ceramic-based dental restorations are of particular importance due to their superior biocompatibility and aesthetics compared to porcelain fused to metal restorations [1]. More specifically, 3 mol% yttria-stabilized tetragonal zirconia polycrystalline (3Y-TZP) has become one of the main substitutes for metal ceramic restorations because of its high flexural strength and fracture toughness, in addition to its low enamel and material wear [2]. However, zirconia exhibits a lower translucency and a high opacity compared to lithium disilicate ceramics, thereby resulting in inferior aesthetics [3].

To improve the material translucency and overcome this shortcoming, newer generations of zirconia with higher translucency parameters have been developed. More specifically, first-generation materials consisted of 3Y-TZP and contained 0.25 wt% alumina (Al_2_O_3_) [4]. Although the strength and fracture toughness of this generation of materials were higher than those of newer generations, they exhibited a high opacity due to the presence of tetragonal zirconia phases, which resulted in light scattering from the grain boundaries, additive inclusions, and pores [4]. This type of zirconia is therefore recommended for the fabrication of framework materials in fixed partial dentures and porcelain-layered crowns. The second generation of 3Y-TZP materials was characterized by a reduced porosity that was achieved using a higher firing temperature and by reducing the amount of alumina within the material [5,6]. Such improvements resulted in a 3Y-TZP material with a higher translucency, which is otherwise known as highly translucent zirconia. Although the second generation of 3Y-TZP materials showed an improved translucency, their aesthetics were insufficient for use as monolithic ceramic restorations in the anterior aesthetic zone [4].

Subsequently, third generation materials were developed using an yttria content of 5 mol% (5Y-ZP), which is referred to by manufacturers as ultra-translucent (or super-high-translucent) zirconia, while fourth generation materials were based on zirconia with an yttria content of 4 mol% (4Y-ZP), which exhibited enhanced mechanical properties [7]. Both the third and fourth generation materials contained increased amounts of the cubic phase, which imparted the material with its superior translucency [4]. Such materials therefore become suitable for use in the fabrication of anterior crowns and fixed partial dentures [4]. Due to the increased amounts of the cubic phase present in the newer generation materials, stress-induced transformations do not take place, ultimately resulting in a marked decrease in their strength and fracture toughness properties [8,9,10]. Bonding of zirconia to tooth structure presents a clinical problem as zirconia cannot be etched with acids as in glass-based systems. A previous meta-analysis reported that a combination of mechanical and chemical surface pre-treatment processes is essential to obtain durable bonding to zirconia [11].

To alter the internal surface of zirconia and increase both its mechanical retention and its bonding to the resin cement, air abrasion is commonly employed [12] using airborne particles such as aluminum oxide [13]. The air abrasion of 3Y-TZP results in the formation of a compressive layer that acts as a protective surface, and this can be attributed to a transformation toughening from the tetragonal to the monoclinic phase [14]. Moreover, it leads to the formation of surface flaws that limit the strength owing to crack propagation [14]. A further study reported that the use of an air particle abrasion pressure of 0.2 MPa for this process resulted in a more stable and reliable bond strength between the resin cement and three zirconia materials with varying translucency values [15].

However, the effect of air particle abrasion on the flexural strength of 3Y-TZP zirconia is controversial, since it depends on the size of the abrading particles, their type, and the air pressure employed [12]. For example, some studies have reported an increased flexural strength after air particle abrasion, and this could be accounted for by considering that the transformation toughening mechanism counterbalances any potential critical defect introduced by airborne particle abrasion [16,17,18,19,20]. In contrast, other works have reported a decreased strength after air particle abrasion owing to the introduction of large surface flaws [21,22,23].

Currently, limited evidence is available regarding the use of low abrasive particles and low air pressures when treating high-translucent zirconia [24]. Air abrasion on tooth enamel, dentin, and nickel-chromium alloys with glass beads is commonly used [25,26], which results in low bond strength values for nickel–chromium alloys when compared with aluminum oxide treatment [25]. In addition, air abrasion with glass beads has been found to decrease the bonding strengths of materials to enamel and dentin, whereas air abrasion with alumina decreased the adhesion to enamel but not to dentin [26]. Due to the fact that glass beads are softer than alumina particles, they could be considered an alternative surface treatment for high-translucent zirconia [27].

To date, the effects of different air particle abrasion protocols on the flexural strength of highly translucent zirconia have yet to be examined in detail. The aim of this study is therefore to evaluate the biaxial flexural strength (BFS) values and carry out a qualitative fractographic analysis of high-translucent and ultra-translucent monolithic zirconia ceramics subjected to different mechanical surface treatment protocols. Two null hypotheses are tested: (1) the biaxial flexural strengths of high-translucent and ultra-translucent zirconia ceramics are not affected by air abrasion surface treatment with 50 µm aluminum oxide particles, and (2) the BFSs of high-translucent zirconia and ultra-translucent zirconia ceramics are not affected by air abrasion surface treatment with 50 µm glass microbead particles.

## 2. Materials and Methods

### 2.1. Preparation of the Samples

Table 1 lists the various materials employed in this study along with their compositions and commercial sources.

**Figure 1:**
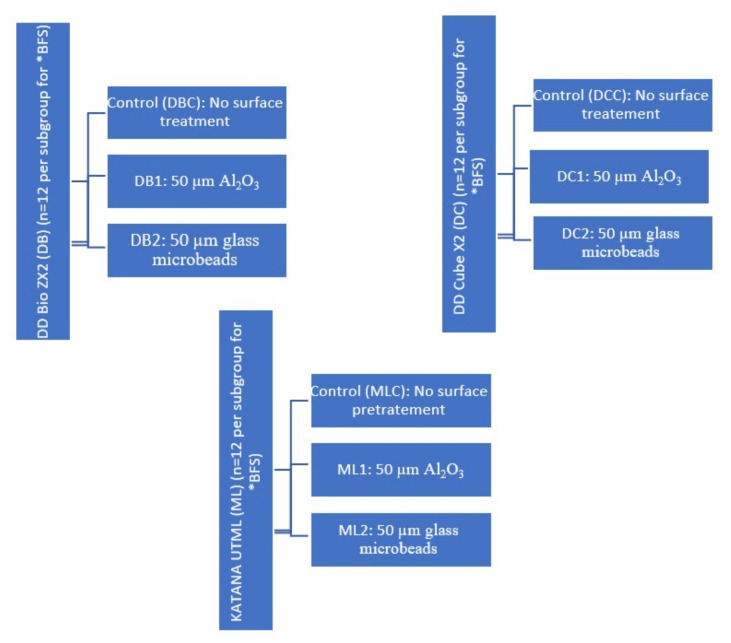
Schematic representation of the different ceramic material groups to be subjected to surface pre-treatment with different types of air abrasion particles. These specimens were then employed in the biaxial flexural strength test. * DB: DD Bio ZX2 ceramic material, DC: DD Cube X2 ceramic material, ML: KATANA UTML ceramic material, BFS: biaxial flexural strength.

A total of 108 zirconia discs (15 mm diameter, 1.5 mm thickness) were prepared from pre-sintered zirconia blocks (KATANA Zirconia UTML, Kuraray Noritake Dental, Tokyo, Japan; DD Bio ZX2, Dental Direkt Materials, Germany; and DD Cube X2, Dental Direkt Materials, Germany) using computer-aided design and computer-aided-manufacture techniques (DWOS, Dental Wings, Montreal, QC, Canada) [28]. The sintering of all samples was performed according to the manufacturer’s recommendations, as outlined in Table 2. Both sides of each sample were polished with 600 and 1200 grit silicon carbide paper under wet conditions for 15 s [15]. The samples were ultrasonically cleaned in distilled water for 10 min and then air-dried prior to surface treatment. The samples were subsequently divided into nine subgroups (*n* = 12 per group) according to the surface treatment employed (Figure 1). More specifically, the control group underwent no surface treatment; the DB1, DC1, and ML1 subgroups were subjected to air abrasion with 50 µm aluminum oxide particles; and the DB2, DC2, and ML2 groups were subjected to air abrasion with 50 µm glass microbeads.

Each air abrasion procedure was performed under a standardized pressure of 2 bar with a nozzle placed at a 90° incidence angle from the center of the sample. The nozzle was placed at a distance of 10 mm and air abrasion was carried out for 20 s using a sandblaster (Duostar Plus, BEGO, Germany) [15,29,30].

### 2.2. Biaxial Flexural Strength (BFS)

A total of 108 samples were ultrasonically cleaned with 99% isopropanol for 180 s, air-dried, and subjected to biaxial flexural strength testing according to ISO 6872 [31]. The samples were placed on three steel balls measuring 3.2 mm in diameter, which were in turn placed on a 10 mm diameter support circle with an angle of 120° between the steel balls. A mechanical testing machine (Type 5567, Instron, Canton, MA, USA) was employed to impart a 5 kN load onto the sample with a crosshead speed of 0.5 mm/min until sample fracture occurred. The load was applied using a piston-shaped indenter with a diameter of 1.2 mm, and the load was directed to the center of the sample. The BFS was measured according to ISO 6872 [28,30,31] and was calculated as follows:

S = −0.2387P (X − Y)/d^2^

X = (1 + v)In(r_2_/r_3_)^2^ + ([1 − v]/2) (r_2_/r_3_)^2^

Y = (1 + v) (1 + In[r_1_/r_3_]^2^) + (1 − v)(r_1_/r_3_)^2^

where S is the BFS (MPa), P is the fracture load (N), d is the disc specimen thickness at the fracture site (mm), v is Poisson’s ratio (0.25), r_1_ is the radius of the support circle (5 mm), r_2_ is the radius of the loaded area (0.6 mm), and r_3_ is the radius of the specimen (6 mm).

### 2.3. Fractographic Analysis

The fractured surfaces were ultrasonically cleaned for 10 min using distilled water, then air-dried and examined using a digital microscope (DIGITAL MICROSCOPE KH-7700, Hirox, Tokyo, Japan) for determination of the fracture origin in relation to the fractography principles of ceramics [32]. The selected representative fractured samples were evaluated by scanning electron microscopy (JSM-6360LV, JEOL Ltd., Tokyo, Japan) with magnification ranges from 50× to 400×. The samples were coated with a thin coating of gold via ion sputtering (JFC-1100, JEOL Ltd., Tokyo, Japan). The fracture origins were determined on the fracture surface by recognizing specific fracture patterns, tracing back to the fracture origin sites, and examining the progression of fracture. A number of fracture patterns were identified in the descriptive fractographic analysis of fractured zirconia samples, including hackles, twist hackles, arrest lines, compression curls, and void defects [33].

### 2.4. Statistical Analysis

Data were collected and grouped for statistical analysis using a statistical software package (SPSS version 23). Statistical analysis was performed using the Shapiro–Wilk test of normal distribution (*p* > 0.05). Two-way analysis of variance (ANOVA) was conducted to evaluate the null hypothesis, followed by Scheffe’s post hoc tests for multiple comparisons (*p* < 0.05). The level of significance was set at *p* ≤ 0.05.

## 3. Results

### 3.1. Biaxial Flexural Strength (BFS) Testing

Two-way ANOVA showed a statistically significant difference in the BFS between the various surface pre-treatment groups (*p* < 0.05), and the interaction effect between the ceramic material group and the surface pre-treatment group was also found to be statistically significant (*p* < 0.05). In addition, one-way ANOVA showed a statistically significant difference between the BFS values of the surface pre-treatment groups for all ceramic materials (*p* < 0.05) (Table 3).

**Figure 2:**
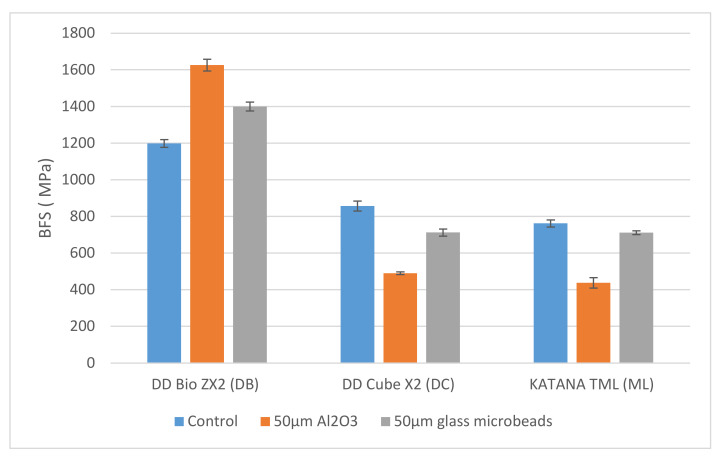
Mean biaxial flexural strengths (MPa) of the various experimental groups.

The mean ± SD BFS for the DD Bio ZX2 (DB) sample reached its highest value after treatment with 50 µm Al_2_O_3_, with a lower value being obtained following treatment with 50 µm glass microbeads, and the lowest value being observed for the control. In contrast, the mean ± SD BFSs for the DC and ML samples reached their highest values in the control and became lower after treatment with the 50 µm glass microbeads and the 50 µm Al_2_O_3_ particles (Figure 2).

Scheffe’s post hoc test showed that for the DB, DC, and ML materials, statistically significant differences were present in the BFS values between the various sample groups (*p* < 0.05). More specifically, for the DB samples, the mean ± SD BFS after treatment with 50 µm Al_2_O_3_ was significantly higher than the control sample and after treatment with the 50 µm glass microbeads (*p* < 0.05). In contrast, both the DC and ML samples showed mean ± SD BFS values that were statistically significantly higher in the control group than after treatment with either the 50 µm Al_2_O_3_ beads or the 50 µm glass microbeads (*p* < 0.05) (Table 4).

### 3.2. Fractographic Analysis

**Figure 3:**
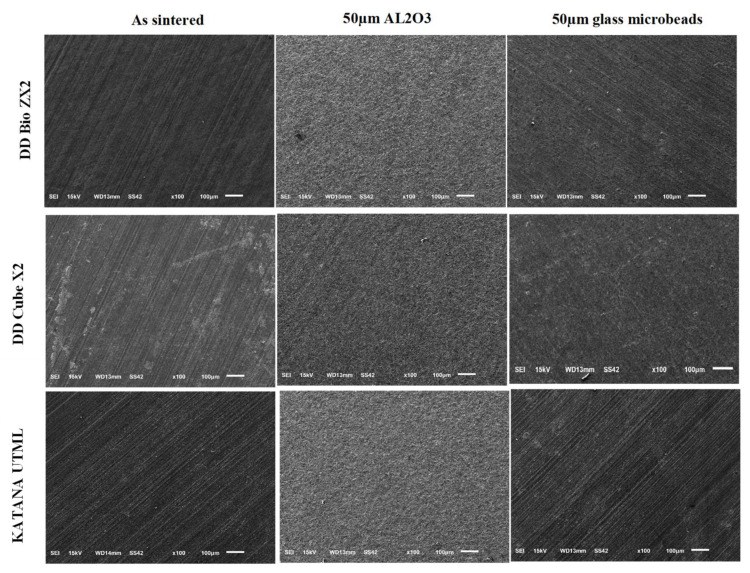
Representative SEM images showing the effects of abrasion using 50 µm aluminum oxide particles and 50 µm glass microbeads on the surfaces of different zirconia ceramics.

**Figure 4:**
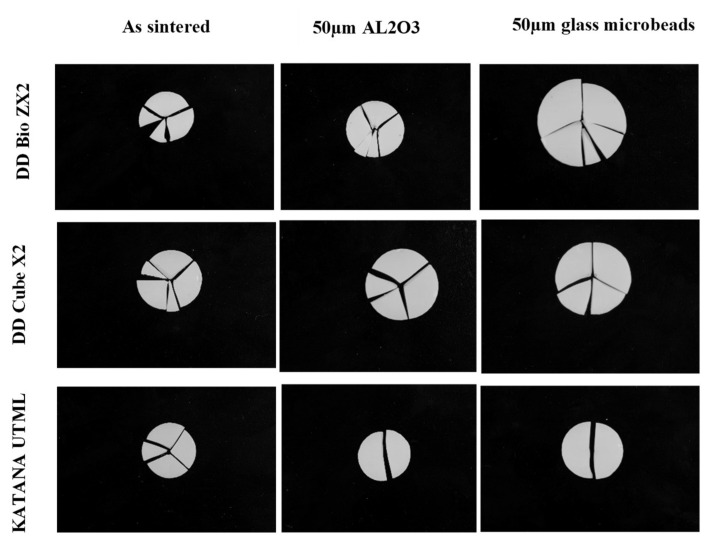
Representative fractured discs for the different zirconia groups.

Significant differences in the topography of the abraded ceramic surfaces in comparison with control can be seen (Figure 3). The use of 50 µm Al_2_O_3_ particles resulted in the generation of rougher surfaces, while for the groups treated with 50 µm glass microbeads, pitting of the surface was observed in all cases. The various fractured discs obtained following surface treatment of the samples are shown in Figure 4. Compared to the sintered control group, it can be seen that the airborne particle-abraded DB samples shattered into multiple fragments of varying sizes, while fewer fragments were obtained for the airborne particle-abraded DC and ML samples.

**Figure 5:**
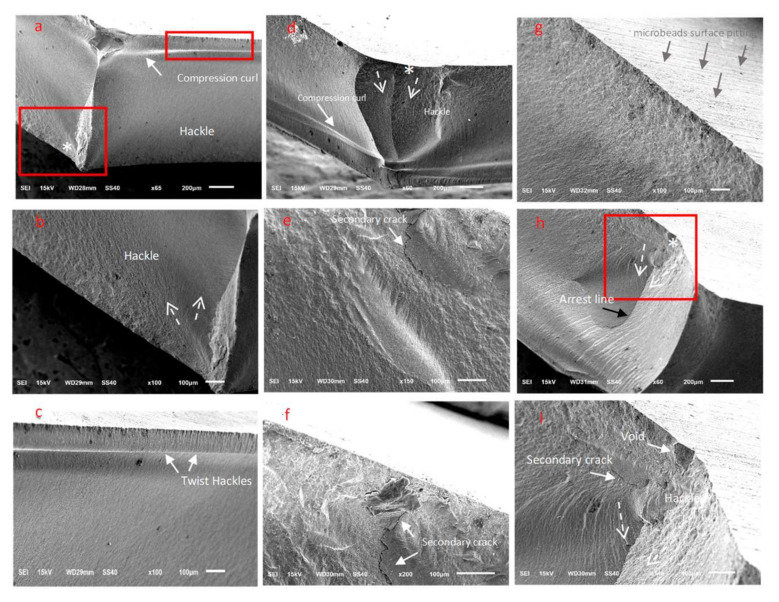
Representative SEM images of the ceramic DB fractured discs. (**a**–**c**) The control as sintered group, (**d**–**f**) the 50 µm Al_2_O_3_-treated group, and (**g**–**i**) the 50 µm glass microbead-treated group.

**Figure 6:**
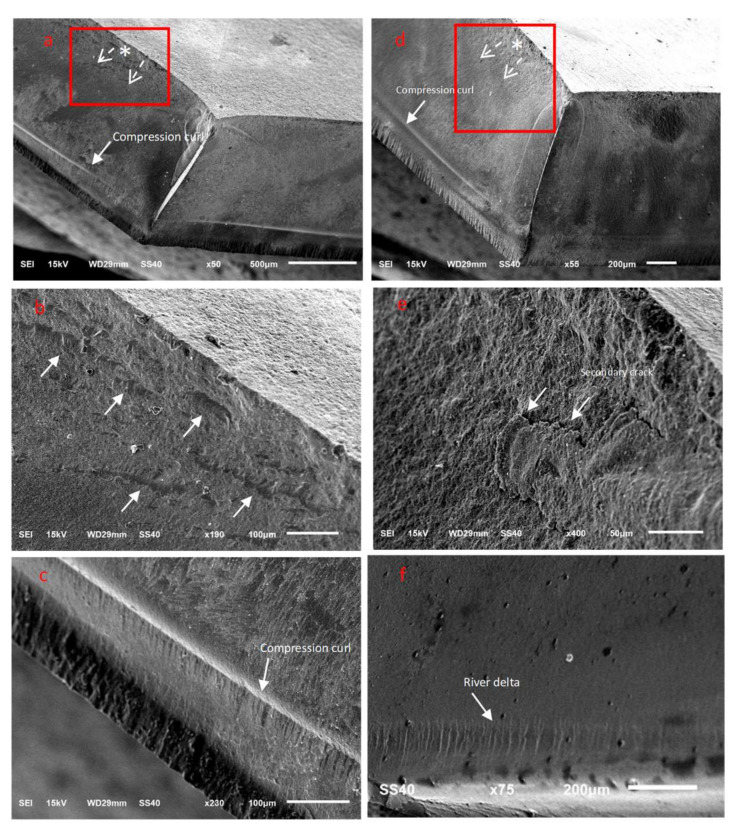
Representative SEM images of the ceramic DC fractured discs. (**a**–**c**) The 50 µm Al_2_O_3_-treated group, and (**d**–**f**) the 50 µm glass microbead-treated group.

**Figure 7:**
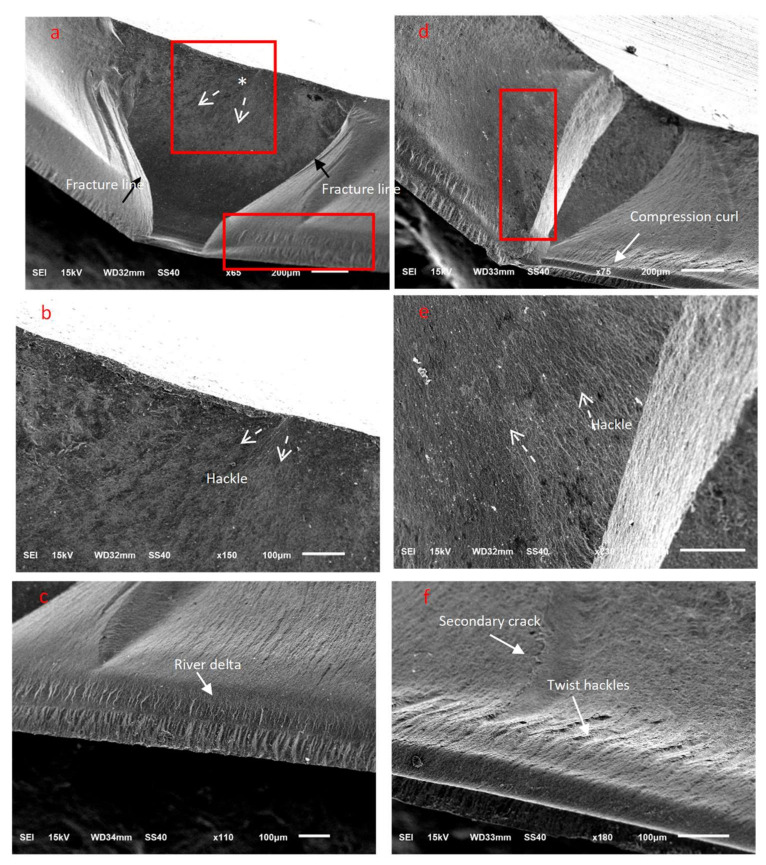
Representative SEM images of the ceramic ML fractured discs. (**a**–**c**) The 50 µm Al_2_O_3_-treated group, and (**d**–**f**) the 50 µm glass microbead-treated group.

SEM examinations revealed numerous characteristic features for the fractured zirconia ceramic surfaces, wherein the fracture origins (*), direction of crack propagation (dotted arrows), secondary cracks, hackles, river delta, arrest lines, and compression curls were clearly evident (Figure 5, Figure 6 and Figure 7). Twist hackles formed when the principal tension axis undergoes a lateral rotation and the crack twists (Figure 5c). As the crack approaches the compression side of the sample, the crack slows down to leave a curved lip (compression curl) just before the total fracture of the material indicating the end of the fracture (Figure 5d, Figure 6c, and Figure 7d). An arrest line can also be observed (black arrow), indicating a change in direction of the crack propagation (Figure 5h). The DB sample tended to exhibit a smoother fractured surface than the other tested zirconia materials, although multiple secondary cracks were also present, which led to the generation of smaller ceramic fragments (Figure 5). For the DC sample, cracks began to appear at areas that exhibited surface and subsurface porosity, wherein the cracks progressed in multiple planes (Figure 6b), and secondary cracks were frequently observed in the fractured specimens (Figure 6). Furthermore, for the ML sample, clear fracture lines and smooth fracture surfaces were observed (Figure 7).

## 4. Discussion

In the present study, we evaluated the BFSs of three highly translucent monolithic zirconia ceramics after subjecting them to different mechanical surface treatment protocols. Previous studies have demonstrated that sandblasting with aluminum oxide particles at 0.2 MPa improved the BFSs of both conventional and high-translucent 3Y-TZP zirconia ceramics [34,35,36]. In our study, a similar result was observed for the DB high-translucent zirconia ceramic, which showed an increased flexural strength following air abrasion with both aluminum oxide and glass microbeads. However, we found that air abrasion decreased the BFS values for both DC and ML zirconia ceramics. Thus, the null hypothesis was rejected since it was found that the BFS was affected by the use of aluminum oxide or glass microbead air abrasion surface treatments.

The flexural strength of dental ceramics can be determined using 4-point and 3-point bending tests or a BFS test [37]. However, it should be noted that the fabrication of samples for uniaxial flexure testing methods can produce defects or flaws within the edges of samples, which can result in great variations in their resulting strength values when subjected to a load [35]. In contrast, the BFS test is advantageous for preventing premature failures from such flaws or cracks because these defective areas are not subjected directly to the load [38]. Moreover, strength tests using multiaxial loading could mimic the loading during the mastication process and so could be considered beneficial for examining the relevant mechanical properties of brittle dental materials [39]. Thus, the BFS test was employed for the purpose of this study. Previously, BFS tests have been carried out using different types of load configurations, such as ball on three balls, ball-on-ring, ring-on-ring, and piston-on-three-ball biaxial loadings [40,41]. For the piston-on-three-ball test, the load is applied by a piston at the center of the disc-shaped sample, which is supported by three metallic balls [31,37]. Although the piston-on-three-ball test can produce a non-uniform stress distribution under the piston, the strain will be increased to a greater degree than what is expected within the sample [42], and this is the only test that has been selected by the International Organization for Standardization (ISO 6782) for testing the BFS of dental ceramic materials [31]. Therefore, the piston-on-three-ball BFS test was implemented for the purpose of this study.

Air abrasion by alumina particles is known to promote a transformation toughening mechanism within the zirconia, in which the tetragonal to monoclinic phase transformation is accompanied by a volumetric expansion that limits the propagation of the initiated cracks [6,37,43]. This transformation toughening mechanism releases a residual compressive stress on the surface, which results in an increase in the fracture toughness and the BFS of the high-translucent 3Y-TZP material [44,45]. This could explain the high BFS values observed for the DB ceramic when subjected to airborne particle abrasion with either 50 µm aluminum oxide or 50 µm glass microbeads. Similar to our findings, a previous study evaluated the effects of different surface treatments, including surface abrasion with 50 µm alumina particles, and concluded that such treatment methods increased the BFSs of conventional and high-translucent 3Y-TZP zirconia [34,35]. In contrast, reduced BFS values were observed for both 5Y-TZP DC and ML zirconia ceramics when the surfaces were treated with either aluminum oxide or glass microbeads. These results were in agreement with a previous study that reported a negative correlation between the BFS and the translucency of zirconia [37]. It should also be noted here that transformation of the tetragonal phase to the monoclinic phase within zirconia is highly dependent on the yttria content and the ceramic microstructure [46]. Thus, the increased yttria content of the 5Y-TZP ceramics resulted in a lower transformability of the tetragonal phase and hence a reduced release of compressive stresses and a decreased flexural strength [44,46]. In addition, the decreased strength was attributed to the lack of transformation toughening with an increased content of the cubic phase in these high-translucent zirconia ceramics [9]. Furthermore, it was previously reported that the mechanical stress caused by airborne particle abrasion could promote rhombohedral phase transformation in high-translucent zirconia, thereby resulting in lower mechanical properties [47]. A recent study also evaluated the effects of the alumina particle size on the release of residual compressive stresses from conventional and high-translucent 5Y-ZP zirconia ceramics, and as a result, the use of 110 µm aluminum oxide particles was suggested to treat the 3Y-TZP zirconia surface, while a particle size of only 25 µm was suggested for 5Y-ZP zirconia ceramics [48].

Fractography allows for the accurate examination of fractured surfaces that contain microscopic features in which the direction of crack propagation is pointing toward the fracture origin site [49]. The qualitative fractography technique used in this study involves a description of the microscopic surface and the subsurface fracture features that indicate the crack origin site and the direction of crack propagation within each ceramic material. Various characteristic ceramic fracture features, such as hackles, twist hackles, fracture origins, arrest lines, and compression curls, were evident in the tested translucent zirconia fractured discs. When the maximum velocity of the crack is reached during crack propagation, a secondary crack hackle line is formed, and this line runs parallel to the direction of crack propagation and perpendicular to the crack origin site [33]. Twist hackles separate portions of the crack surface in which they rotate from the principal crack plane as a result of twisting in the principal tension axis [33]. Another characteristic feature is an arrest line, in which a well-defined sharp line occurs when the crack changes its direction while propagating, and this could be helpful in indicating the failure origin [50]. When the discs were loaded for flexural testing, a crack was initiated and propagated perpendicular to the tensile surface; this represents the crack origin site. As the crack propagates, it approaches the compression side of the sample and slows down, causing a compression curl to form, which is a common feature in ceramic failure and indicates the end of the fracture [50]. The presence of a compression curl is an important feature, since it indicates that the sample possessed a bending component when loaded [33]. It should be noted here that the fracture origin is usually located on the side opposite to that of the compression curl [33,50].

We found that the DB sample exhibited multiple secondary cracks that likely led to shattering of the ceramic disc into multiple fractured pieces of varying sizes and the loss of smaller fragments of ceramics. For the DB sample, the resulting surface and subsurface porosities promoted crack initiation in these areas, and progress in multiple planes was frequently observed wherein secondary cracks were also present. Furthermore, in the case of the ML sample, a smooth fracture surface with clear fracture lines was generally observed.

Thus, based on the results of this study, the use of 50 µm aluminum oxide particles for air abrasion at 0.2 MPa pressure could be recommended for the surface treatment of high-translucent 3Y-TZP zirconia ceramics. In the case of 5Y-ZP ceramics, the use of 50 µm glass microbeads for surface treatment negatively affected the BFS values, and so a smaller particle size should be considered in further studies.

The limitations of the current study include the fact that we concentrated on the type of air abrasion particles, while all other parameters were maintained constant. A limitation of the methodology used in this research is that the samples were machined, leaving micro-grooves that could affect the transformation at the surface, and this could impact durability and strength of zirconia. Moreover, the effect of aging was not examined. Future studies should therefore investigate additional parameters, such as different air abrasion pressures and particle sizes.

## 5. Conclusions

Within the limitations of this study, it could be concluded that the air abrasion of a 3 mol% yttria-stabilized tetragonal zirconia polycrystalline (3Y-TZP) translucent zirconia surface with 50 µm Al_2_O_3_ particles at a pressure of 2 bar resulted in the highest BFS values. In addition, the BFS of the tested 5Y-ZP ultra-translucent zirconia ceramic was negatively affected when air abrasion was carried out using either 50 µm Al_2_O_3_ particles or 50 µm glass microbeads. Smaller particle size should be considered in future research.

## Figures and Tables

**Table 1:** Materials employed during this study.

Material	Brand Name	Shade	Composition	Manufacturer
High-translucent 3Y-TZP ceramic	DD Bio ZX2	White	≥99% ZrO_2_ + HfO_2_ + Y_2_O_3_, <6% Y_2_O_3_, ≤0.15% Al_2_O_3_, <1.0% other oxides.	Dental Direkt Materials, Germany
Superhigh-translucent 5Y-TZP ceramic	DD Cube X2	White	≥99% ZrO_2_ + HfO_2_ + Y_2_O_3_, <10% Y_2_O_3_, ≤0.01% Al_2_O_3_, <1.0% other oxides.	Dental Direkt Materials, Germany
Ultra-translucent 5Y-ZP ceramic	KATANA zirconia UTML	White	87–92% ZrO_2_, 8–11% Y_2_O_3_, <2% other oxides.	Kuraray Noritake Dental, Tokyo, Japan
50 µm glass microbead particles	Rolloblast		Glass microbeads	Renfert, Germany
50 µm aluminum oxide particles	Cobra		Aluminum oxide	Renfert, Germany

**Table 2:** Sintering conditions for preparation of the different zirconia materials.

Material (Brand Name)	Sintering Temperature	Holding Time
DD Bio ZX2	1450 °C	9 h
DD Cube X2	1450 °C	9 h
KATANA zirconia UTML	1550 °C	2 h

**Table 3:** Mean (and SD) biaxial flexural strengths (MPa) of the various experimental groups.

		Mean	Standard Deviation	Standard Error	95% Confidence Interval for Mean		Min	Max	*p* Value
					Lower Bound	Upper Bound			
DD Bio ZX2 (DB)	Control	1198.52 ^c^	21.20	6.12	1185.05	1211.98	1162.09	1225.26	0.000 *
	50 µm Al_2_O_3_	1626.06 ^a^	31.94	9.22	1605.76	1646.35	1588.83	1676.23	
	50 µm glass microbeads	1399.53 ^b^	24.25	7.00	1384.12	1414.94	1346.51	1439.88	
DD Cube X2 (DC)	Control	856.73 ^a^	26.89	7.76	839.64	873.82	800.96	923.32	0.000 *
	50 µm Al_2_O_3_	490.40 ^c^	6.96	2.01	485.98	494.83	477.24	501.06	
	50 µm glass microbeads	712.64 ^b^	19.38	5.59	700.32	724.95	687.15	740.27	
KATANA UTML (ML)	Control	761.91 ^a^	19.44	5.61	749.55	774.26	728.21	789.29	0.000 *
	50 µm Al_2_O_3_	437.92 ^c^	29.57	8.54	419.12	456.71	396.21	487.36	
	50 µm glass microbeads	711.76 ^b^	10.39	3.00	705.16	718.36	694.05	730.08	

* Statistically significant at *p* ≤ 0.05. * Different small letters indicate significant differences at *p* ≤ 0.05.

**Table 4:** Scheffe’s post hoc test for the BFS (MPa).

		DD Bio ZX2 (DB)		DD Cube X2 (DC)		KATANA UTML (ML)	
		Mean Difference	*p* Value	Mean Difference	*p* Value	Mean Difference	*p* Value
50 µm Al_2_O_3_	50 µm glass microbeads	226.5240 *	0.000	−222.2337 *	0.000	−273.8480 *	0.000
	Control	427.5403 *	0.000	−366.3253 *	0.000	−323.9907 *	0.000
50 µm glass microbeads	Control	201.0163 *	0.000	−144.0916 *	0.000	−50.1427 *	0.000

* Statistically significant at *p* ≤ 0.05.

## Data Availability

Data sharing is not applicable to this article.
